# Integrating Deep Supervised, Self-Supervised and Unsupervised Learning for Single-Cell RNA-seq Clustering and Annotation

**DOI:** 10.3390/genes11070792

**Published:** 2020-07-14

**Authors:** Liang Chen, Yuyao Zhai, Qiuyan He, Weinan Wang, Minghua Deng

**Affiliations:** 1 School of Mathematical Sciences, Peking University, Beijing 100871, China; clandzyy@pku.edu.cn (L.C.); heqy@pku.edu.cn (Q.H.); wangweinan@pku.edu.cn (W.W.); 2 Mathematical and Statistical institute, Northeast Normal University, Changchun 130024, China; zhaiyy375@nenu.edu.cn; 3 Center for Quantitative Biology, Peking University, Beijing 100871, China; 4 Center for Statistical Science, Peking University, Beijing 100871, China

**Keywords:** single-cell RNA sequencing, clustering and annotation, supervised learning, self-supervised learning, unsupervised learning

## Abstract

As single-cell RNA sequencing technologies mature, massive gene expression profiles can be obtained. Consequently, cell clustering and annotation become two crucial and fundamental procedures affecting other specific downstream analyses. Most existing single-cell RNA-seq (scRNA-seq) data clustering algorithms do not take into account the available cell annotation results on the same tissues or organisms from other laboratories. Nonetheless, such data could assist and guide the clustering process on the target dataset. Identifying marker genes through differential expression analysis to manually annotate large amounts of cells also costs labor and resources. Therefore, in this paper, we propose a novel end-to-end cell supervised clustering and annotation framework called scAnCluster, which fully utilizes the cell type labels available from reference data to facilitate the cell clustering and annotation on the unlabeled target data. Our algorithm integrates deep supervised learning, self-supervised learning and unsupervised learning techniques together, and it outperforms other customized scRNA-seq supervised clustering methods in both simulation and real data. It is particularly worth noting that our method performs well on the challenging task of discovering novel cell types that are absent in the reference data.

## 1. Introduction

The rapid development of single-cell RNA sequencing technologies allows us to study tissue heterogeneity at the cellular level [1,2]. Cell clustering and annotation are two fundamental steps in analyzing single-cell RNA-seq data [3]. For example, exploring cell-to-cell interactions and gene-to-gene interactions are often based on specific cell types [4,5]. In the past few years, a large volume of single-cell transcriptome unsupervised clustering algorithms have emerged based on the similarity of gene expression patterns [6,7,8,9]. However, in the absence of a unified standard, the clustering results of different algorithms usually show a little degree of overlap and even vary widely [10]. The typical cell annotation method finds differential expression genes between the identified clusters [11] and then annotates cells by leveraging the function of these differential expression genes [12]. However, the process can be complicated by the low sensitivity of the well-known marker genes. In addition, as the number of mapped cells increases, such as the Human Cell Project (HCA) [13] and Mouse Organogenesis Cell Atlas (MOCA) [14], this method of cell annotation also becomes a highly labor-intensive task.

Recently, researchers have tried to use prior information to guide cell clustering and annotation on the target dataset (see Table 1). For example, Zhang et al. proposed CellAssign, a probabilistic model that leverages prior knowledge of cell type marker genes to annotate single-cell RNA sequencing data into predefined or de novo cell types [15]. Similarly, Pliner et al. proposed Garnett, a tool for rapidly annotating cell types in single-cell transcriptional profiling datasets, based on an interpretable, hierarchical markup language of cell type specific marker genes [16]. However, these two methods require users to provide a list of marker genes corresponding to the cell types in advance. For non-experts, this is not a trivial matter, because ample knowledge of the marker genes usually requires a large amount of literature review and long-term accumulation.

With more and more large-scale, well-annotated datasets becoming available, it is feasible to apply supervised classification techniques for categorizing cells into known cell types [17,18]. For example, SingleR performs cell type classification by calculating the Spearman correlation between query cells and one representative gene expression per cell type in reference datasets [19]; Moana classifies cells in the target data by using a support-vector-machine (SVM) with a linear kernel that is well-trained on the labeled source data [20]. In this way, much time and effort are saved in matching marker genes. However, although classification techniques do not require domain knowledge of the cell types, they usually require the reference dataset to cover all cell types contained in the target dataset, thus limiting the ability to discover novel cell types. Similar problems also arise in mapping strategies. To illustrate, scmapCluster assigns cell types by calculating the maximum similarity between unannotated cells and the cluster centers of well-annotated cell types in the reference dataset [21]. Although scmapCluster can predict certain cells as "unassigned" when the similarity score is lower than a predefined threshold, subjectively selecting threshold parameters, coupled with the failure to consider the global structure of clusters, usually makes the annotation results less satisfactory. In addition, technical biases, such as batch effects, also invalidate these projection algorithms [22].

Deep learning has achieved impressive performance over traditional machine learning algorithms on many bioinformatics tasks over the past few years [23]. Lopez et al. developed a ready-to-use scalable framework, scVI, for the probabilistic representation and analysis of gene expression in single cells [24]. Xu et al. developed scANVI, a semi-supervised variant of scVI designed to leverage any available cell state annotations for annotating new datasets [25]. However, the extra latent space assumption like mixture Gaussian distribution cannot perfectly portray the low-dimensional manifolds where data resides most of the time. Moreover, variational inference requires advanced optimization techniques; otherwise, the objective function can easily fall into local extreme values. Hu et al. developed ItClust, an iterative transfer learning algorithm with deep neural network for scRNA-seq clustering [26]. ItClust learns cell type knowledge from well-annotated source datasets, but it also leverages information in the target dataset to make it less dependent on the quality of the source dataset. Nevertheless, transfer learning based on cluster centers is easily infected by batch effects, resulting in false cell type annotations. When the target dataset holds the cell types that do not exist in the source dataset, it is also difficult for ItClust to peform accurate discovery. Furthermore, neither scANVI nor ItClust considers the affinity constraint of pairwise similar cells, which can often improve the effect of cell clustering and annotation [7].

In this paper, we combine deep learning technique with statistical modeling and propose an end-to-end single-cell RNA-seq data supervised clustering and annotation framework, named scAnCluster, based on our previous unsupervised clustering works scDMFK [6] and scziDesk [7]. The three methods adhere to similar modeling concepts, namely simultaneous denoising, dimensionality reduction and clustering. The difference is that the main purpose of scAnCluster is to implement automatic cell type annotation based on clustering. In addition to transferring cell type knowledge, scAnCluster also needs to balance the importance of the label information on the reference data and the unique characteristics of the target data. In this regard, we dismember this semi-supervised learning task as the integration of supervised learning, self-supervised learning and unsupervised learning. First, we model the count data distribution in the mixed annotated source dataset and unannotated target dataset. Then we use the deep autoencoder to learn the low-dimensional manifold where the distribution parameters are located, giving us the joint latent representation of different datasets. Second, we learn a classifier on the well-labeled data to enhance and aggregate each known cell type, at the same time equipping the latent space with basic cluster recognition ability. Third, we calculate the pairwise similarity among all cells and obtain pairwise pseudo-labels by determining whether each cell pair is similar or not, thereby constructing a pairwise classification task to fuse labeled and unlabeled cells. This step uses a meta-learning technique and can be regarded as a proxy for clustering, which also provides guidance for subsequent clustering. Finally, we use an iterative soft k-means model with entropy regularization based on spherical distance to perform clustering on the mixed datasets. After that, we can assign known cell types in the source dataset to those clusters with a high confidence level. In order to prevent overfitting and destroying the global structure of the data, our training process carries the decoder for data reconstruction and denoising from beginning to end. In addition, our model only requires users to provide the total cluster number of the joint datasets, and it imposes no constraints or restrictions on the number of clusters for the target dataset to be annotated.

## 2. Materials and Methods

### 2.1. Zero-Inflated Negative Binomial Distribution for scRNA-seq Data Modeling

Assume a single-cell RNA-seq data matrix is $x_{n\times m}$ where *n* represents total cell number, and *m* refers to the gene feature number. Furthermore, *x* includes the source dataset matrix $x_{n_{1}\times m}^{s}$ and target dataset matrix $x_{n_{2}\times m}^{t}$. The source dataset is well labeled with cell type number $k_{s}$, and its label set is $\left\{y_{i}^{s},1\leq i\leq n_{1}\right\}$. Our goal is to automatically cluster the unlabeled target dataset by mixing two datasets together and leveraging the known label information in the source dataset. We construct the one-hot encoded batch matrix $b_{n\times 2}$ where its non-zero element position in each row $b_{i}$ represents the corresponding batch index, namely source dataset and target dataset. Since the mean of gene expression data is usually larger than its dispersion [27], we assume that this discrete count data follow negative binomial distribution (NB), as

(1) $$\begin{array}{cc} p_{nb}\left(x_{ij}|b_{i}\right) & =NB(x_{ij}|\mu_{ij},\theta_{ij},b_{i}) \end{array}$$

(2) $$\begin{array}{cc} \; & =\frac{\Gamma (x_{ij}+\theta_{ij})}{\Gamma \left(x_{ij}+1\right)\Gamma \left(\theta_{ij}\right)}{\left(\frac{\theta_{ij}}{\theta_{ij}+\mu_{ij}}\right)}^{\theta_{ij}}{\left(\frac{\mu_{ij}}{\mu_{ij}+\theta_{ij}}\right)}^{x_{ij}} \end{array}$$

Based on the low sensitivity of massive genes, namely “dropout” events, we use mass density $\pi_{ij}$ to account for dropout probability of the *j*-th gene in the *i*-th cell, thereby making data distribution a natural mixture distribution, i.e., zero-inflated negative binomial distribution (ZINB), as

(3) $$\begin{array}{cc} p_{zinb}\left(x_{ij}|b_{i}\right) & =ZINB(x_{ij}|\pi_{ij},\mu_{ij},\theta_{ij},b_{i}) \end{array}$$

(4) $$\begin{array}{cc} \; & =\pi_{ij}\delta_{x_{ij}=0}+\left(1-\pi_{ij}\right)*p_{nb}\left(x_{ij}|b_{i}\right) \end{array}$$

Complex gene-to-gene interactions do not completely free the parameter sets $\left\{\pi_{ij},\mu_{ij},\theta_{ij}\right\}\left(1\leq i\leq n,1\leq j\leq m\right)$ from one another. Furthermore, they are actually located on an inenarrable low-dimensional manifold. Therefore, we use the deep autoencoder representation to approximate this parameter space and estimate three groups of parameters by three output layers in a manner similar to that of the DCA and scziDesk model [7,28]. To take the batch effects into account, we merge the expression data matrix *x* with batch matrix *b* as the input of the encoder network. Similarly, we also concatenate the latent space representation matrix *z* and batch matrix *b* as the input of the decoder network to output the estimation of batch-related parameters $\left\{\pi_{ij},\mu_{ij},\theta_{ij}\right\}\left(1\leq i\leq n,1\leq j\leq m\right)$. This approach is interpretable because it is equivalent to treating the batch effect as a covariate and using the nonlinear regression capability of the neural network to eliminate this part of the deviation. Finally, we take the negative log-likelihood function of ZINB distribution as data reconstruction loss, as

(5) $$\begin{array}{cc} L_{1}\left(\pi ,\mu ,\theta |x,b\right) & =-\sum_{i=1}^{n}\sum_{j=1}^{m}\ln p_{zinb}\left(x_{ij}|b_{i}\right) \end{array}$$

### 2.2. Supervised Classification on Well-Labeled Source Dataset

Before aligning and integrating the joint low-dimensional representation of the source dataset and target dataset, we should first ensure that cells from the same type in the source dataset are aggregated together [29]. In other words, the latent space representation $z^{s}$ of the cells in the source dataset should be clearly separable. To accomplish this, we connect a classification layer to the last layer of the encoder network. Its node number is the known cell type number in the source dataset $k_{s}$, and its activation function is selected as the softmax function. For convenience, we assume that the one-hot matrix form of label vector $y^{s}$ is ${\tilde{y}}_{n_{1}\times k_{s}}^{s}$ and that the classification prediction probability matrix is $c_{n_{1}\times k_{s}}$. We take the classification loss function as the standard cross-entropy function, which only operates on the well-labeled source dataset.

(6) $$\begin{array}{cc} L_{2} & =-\sum_{i=1}^{n_{1}}\sum_{j=1}^{k_{s}}{\tilde{y}}_{ij}^{s}\ln c_{ij} \end{array}$$

This step actually also ensures that the encoder can map cells in the target dataset that are close to the expression pattern of known cell types into the vicinity of the corresponding cell type location.

### 2.3. Self-Supervised Integration Learning on the Union of Source and Target Dataset

To transfer the cell type label of the source dataset to the unlabeled target dataset, we construct a pairwise-classification task on their union by determining whether the cell pair is similar or not, which can be regarded as the surrogate for clustering [30,31]. Specifically, we first use the joint latent space representation *z* to compute the pairwise similarity matrix *S* measured by the cosine distance, as

(7) $$\begin{array}{cc} S_{ij} & =\frac{z_{i}^{T}z_{j}}{\left|\right|z_{i}{\left|\right|}_{2}\left|\right|z_{j}{\left|\right|}_{2}},1\leq i\leq n,1\leq j\leq n \end{array}$$

Then we iteratively go through a self-supervised meta-learning step to refine these similarities. Technically, we construct a pseudo-labeled matrix by applying dynamic thresholds on a similarity matrix *S*:

(8) $${\hat{R}}_{ij}=\left\{\begin{array}{cc} 1, & \mathrm{if}S_{ij}>u\left(t\right)\\ 0, & \mathrm{if}S_{ij}<l\left(t\right)\\ \mathrm{Not}\;\mathrm{selected}, & \mathrm{otherwise}. \end{array}\right.$$

where i,j∈{1,2,3…,n}. We use the dynamic upper threshold u(t) and lower threshold l(t) to determine whether the cell pair is similar or dissimilar. Furthermore, because we have the gold standard label information on the source dataset, which can be treated as prior knowledge to guide clustering, we can extend the self-labeled matrix $\hat{R}$ as,

(9) $${\hat{R}}_{ij}=\left\{\begin{array}{cc} 1, & \mathrm{if}S_{ij}>u\left(t\right)\;\mathrm{or}\;y_{i}=y_{j}\\ 0, & \mathrm{if}S_{ij}<l\left(t\right)\;\mathrm{or}\;y_{i}\neq y_{j}\\ \mathrm{Not}\;\mathrm{selected}, & \mathrm{otherwise}. \end{array}\right.$$

where *y* is defined as $y^{s}$ on the source dataset and unknown on the target dataset. We then combine this self-labeled matrix $\hat{R}$ with the similarity matrix *S* to compute the self-supervised loss $L_{3}$, as

(10) $$\begin{array}{cc} L_{3} & =\sum_{i=1}^{n}\sum_{j=1}^{n}-{\hat{R}}_{ij}\ln S_{ij}-\left(1-{\hat{R}}_{ij}\right)\ln\left(1-S_{ij}\right) \end{array}$$

It contains items for both the labeled and unlabeled subsets, using the given labels and generated pseudo-labels, thus avoiding the forgetting the issue that may arise with a sequence approach. In the specific implementation, we gradually decrease the u(t) value, while increasing the l(t) value during the training process. This step allows us to gradually select more cell pairs to participate in the similarity fusion training. As the thresholds change, we train our model from easy-to-classify cell pairs to hard-to-classify cell pairs iteratively to pursue and bootstrap the cluster-friendly joint latent space representation. When u(t)<l(t), we stop this iterative similarity fusion process. Most importantly, as the training iterations proceed, target cells that are similar to the cell types in the source dataset can gradually move to the vicinity of where they are located, while those target cells that are dissimilar to source cells would naturally form new diverse populations due to their inherent high similarity in the target dataset.

### 2.4. Unsupervised Soft Clustering Upon the Union of Source and Target Dataset

After self-supervised fusion training, we expected to learn a joint latent space representation *z* suitable for clustering. That is, similar cells are aggregated together and dissimilar cells are separated from each other. Therefore, in the latent space, in order to further enforce cluster compactness, we propose a soft k-means clustering model with entropy regularization to perform cell clustering [32]. Suppose total *k* clusters with cluster centers $v_{i}\left(1\leq i\leq k\right)$; then the optimization objective function for clustering is

(11) $$\begin{array}{cc} \; & \min\sum_{i=1}^{n}\sum_{j=1}^{k}w_{ij}\left|\right|z_{i}-v_{j}\left|\right|+\sigma w_{ij}\ln w_{ij} \end{array}$$

(12) $$\begin{array}{cc} \; & s.t.\forall i,\sum_{j=1}^{k}w_{ij}=1. \end{array}$$

where ||.|| is one kind of distance measurement. In the last section, we used the cosine distance for similarity calculation. Since this soft clustering was found to work well under sphere distance, rather than $L_{2}$ distance in scziDesk or $L_{2,1}$ adaptive distance in scDMFK, between *z* and *v*, we assume that all $z_{i}\left(1\leq i\leq n\right)$ and $v_{j}\left(1\leq j\leq k\right)$ have a unity norm. Then the above clustering model can be re-written as a dot product, as follows:

(13) $$\begin{array}{cc} \; & \min\sum_{i=1}^{n}\sum_{j=1}^{k}2w_{ij}\left(1-z_{i}^{T}v_{j}\right)+\sigma w_{ij}\ln w_{ij} \end{array}$$

(14) $$\begin{array}{cc} \; & s.t.\forall i,\sum_{j=1}^{k}w_{ij}=1. \end{array}$$

In fact, when the *z* and *v* are known, $w_{ij}\left(1\leq i\leq n,1\leq j\leq k\right)$ has a closed form, which is

(15) $$\begin{array}{cc} {\hat{w}}_{ij} & =\frac{\exp(-\frac{2(1-z_{i}^{T}v_{j})}{\sigma })}{\sum_{l=1}^{k}\exp\left(-\frac{2(1-z_{i}^{T}v_{l})}{\sigma }\right)} \end{array}$$

In this paper, the default value of σ is 1. We can see that weight ${\hat{w}}_{ij}$ is a decreasing function of distance between $z_{i}$ and $v_{j}$. We give greater attention to cells close to the cluster center. To a certain extent, ${\hat{w}}_{ij}\left(1\leq i\leq n,1\leq j\leq k\right)$ also gives the membership probability that the *i*-th cell(1≤i≤n) belongs to the *j*-th cluster(1≤j≤k). As such, we can assign cluster labels to each cell according to the maximum membership probability. Finally, we give the following clustering loss for training, as

(16) $$\begin{array}{cc} L_{4} & =\sum_{i=1}^{n}\sum_{j=1}^{k}2{\hat{w}}_{ij}\left(1-z_{i}^{T}v_{j}\right) \end{array}$$

This continuous loss function can benefit from the efficiency of stochastic gradient descent.

### 2.5. Model Training Strategy and Parameter Setting

We summarize our end-to-end clustering model below: denoising autoencoder for estimating data distribution parameters, deep supervised classification for aggregating cells of the same type in the source dataset, deep self-supervised pairwise classification with dynamic similarity threshold for distinguishing highly similar and dissimilar cell pairs, and deep soft k-means clustering with cosine distance for refining clusters. In this paper, we train the whole multi-task model by matching different loss functions in an orderly manner. First, we pretrain the model by uniting $L_{1}$ and $L_{2}$ loss, as

(17) $$\begin{array}{cc} L_{pre} & =L_{1}+L_{2} \end{array}$$

Then we turn to similarity fusion training by combining $L_{1}$ and $L_{3}$ loss, as

(18) $$\begin{array}{cc} L_{fuse} & =L_{1}+\lambda_{1}L_{3} \end{array}$$

where $\lambda_{1}$ is a weight hyperparameter that controls relative balance between two loss parts. Finally, we perform cell clustering training by assembling $L_{1}$ and $L_{4}$ loss, as

(19) $$\begin{array}{cc} L_{fune} & =L_{1}+\lambda_{2}L_{4} \end{array}$$

**Figure 1:**
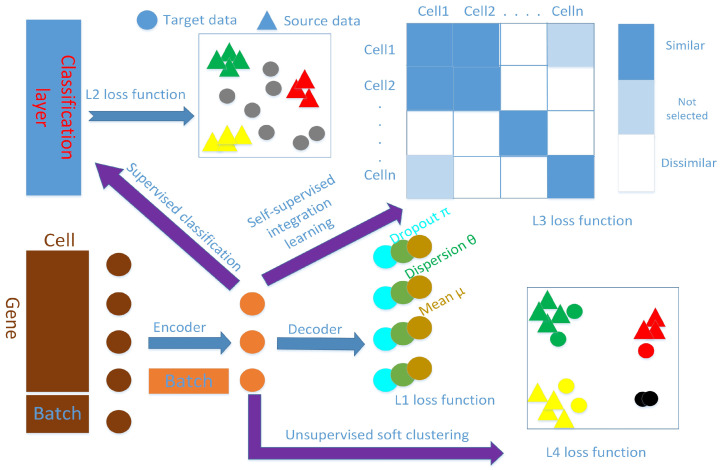
The neural network schematic diagram of scAnCluster. The encoder and decoder are symmetrical structures, and the outputs are three sets of parameters, including dropout rate, mean value and dispersion value, in zero-inflated negative binomial distribution (ZINB) modeling. We concatenate the batch index matrix in the input layer and bottleneck layer. In latent space, the mixed cells will enter three stages of learning in sequence: first the supervised classification learning of cells with known labels, second the self-supervised learning of dynamic similarity fusion of all cells, and third, the unsupervised soft clustering learning of all cells.

Similarly, $\lambda_{2}$ is also a weight hyperparameter. Without any preference, we expect that the contribution of each part of the loss to the gradients is at the same level. In the specific algorithm implementation, $L_{1}$ is averaged over all cells and genes, while $L_{3}$ and $L_{4}$ are averaged over all cells such that $L_{3}$ and $L_{4}$ are naturally larger than $L_{1}$. We use the grid search method to determine these two hyperparameter values from the candidate sets {0.1,0.01,0.001}. Our guiding principle holds that the weights can make the loss function values of both parts the same order of magnitude. Supplementary Table S1 gives their setting in all experiments. We add reconstruction loss $L_{1}$ to each training step because we want to preserve the global structure of the data through it, which can also avoid model overfitting. We implement our method in Python3 with Tensorflow software. The two layers of the encoder are sized as 256 and 64, respectively, and the decoder has the reverse structure of the encoder. The bottleneck layer (namely, the latent space) has a size of 32. The training minibatch size is 256, and the stochastic gradient optimizer is Adam with learning rate 1e-4. The epochs of pretraining and fusion training are 500 and 100, respectively. The dynamic thresholds u(t) and l(t) are set to u(t)=0.95−0.0045t(1≤t≤100) and l(t)=0.455+0.00045t(1≤t≤100), respectively. After similarity fusion training, we take out the latent space representation and use the standard k-means algorithm to obtain cluster centers as the initial values of $v_{i}\left(1\leq i\leq k\right)$. We stop the clustering training when the cluster label assignments no longer change. The schematic diagram is shown in Figure 1, and we name our method scAnCluster. An implementation of scAnCluster is available from https://github.com/xuebaliang/scAnCluster.

### 2.6. Cell Type Annotation for Target Dataset

After completing the clustering, we obtained the cluster label of each cell by the union of the source dataset and target dataset. In order to leverage the cell type information in the source dataset to help annotate each cluster, we define the cluster clarity score. For cluster *i*, we can summarize the cell composition of the source dataset contained in this cluster. When the cell number of a given cell type in the *i*-th cluster is larger than half of its total number in the source dataset, we set it as the candidate reference cell type of the *i*-th cluster and record its corresponding percentage (>0.5). Finally, we take the reference cell type corresponding to the largest percentage as the annotation of the *i*-th cluster, and this largest percentage is regarded as the clarity score of *i*-th cluster. In the meantime, we can also annotate all cells in the target dataset contained in the *i*-th cluster. For those clusters that cannot be annotated through the information of the source dataset, namely "unassigned", we supply their cluster labels, and the user can further apply differential expression analysis and find highly expressed marker genes to annotate these clusters.

### 2.7. Data Preprocessing

All datasets we used have passed through quality control and are in the formats of counts. We unify raw cell type annotation by Cell Ontology [22,33], a structured controlled vocabulary for cell types. We merge the source dataset and target dataset according to their shared gene names. We first normalize the total expression value of each cell to its median value and then take a natural log-transformation on data. Then we select the top 1000 highly variable genes according to their normalized dispersion value ranking. Finally we transform the data into z-score data. That is, each selected gene has zero mean and unit variance. All three steps can be completed using the Scanpy package [34]. We take the preprocessed data as neural network input and use its corresponding original count data for modeling.

## 3. Results

### 3.1. Competing Methods and Evaluation Index

We compare scAnCluster with three other cluster-based scRNA-seq supervised clustering and annotation methods, scmapCluster (recorded as scmap) [21], scANVI [25] and ItClust [26], under their default analytical pipelines and parameters (see Supplementary). For clustering performance evaluation, we use the adjusted rand index (ARI) to compare the clustering labels with true cell type labels on the target dataset. The higher ARI values correlate with better clustering. In addition, we use the annotation accuracy to measure the predicted cell annotations with true cell annotations on the overlapping cell types between the source dataset and the target dataset. Since scANVI only provides cluster labels, we apply our cluster annotation method to it in order to calculate its annotation accuracy. In the next several sections, we will show the superiority of scAnCluster in the simulation datasets, three groups of inter-datasets and one intra-dataset, with partial known well-annotated cells.

### 3.2. Simulation Study

**Figure 2:**
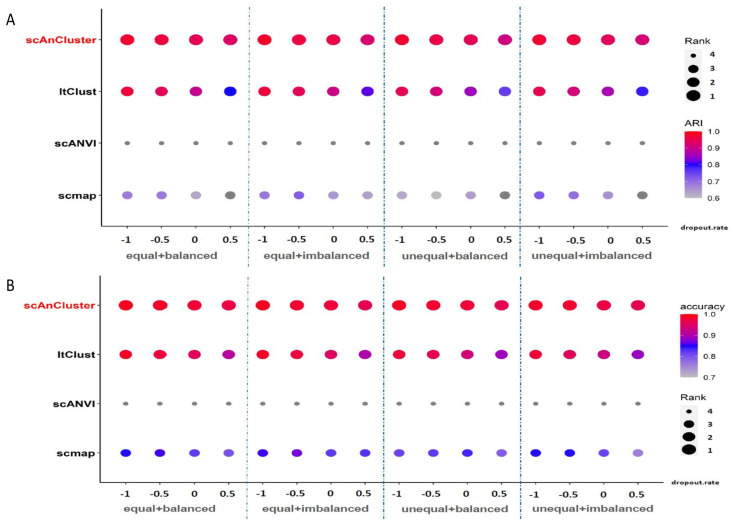
Simulation dataset analysis. (**A**,**B**) Bubble plots of adjusted rand index (ARI) and annotation accuracy values for four methods in sixteen groups of simulation experiments using the whole mixed datasets, respectively.

We used the Splatter package [35] to generate our simulation data, which contains two batches. Each batch initially contains six clusters (the labels can be simply written as 0, 1, 2, 3, 4 and 5), and the number of genes is 2500. For simplicity, we refer to these two batches as the source dataset and the target dataset. The sizes of these two datasets can be equal, that is, “equal” (both 3600 cells), or not equal (the source dataset has 3600 cells; the target dataset has 1800 cells), that is, “unequal”. The cluster sizes in each dataset can be the same, that is, “balanced”, or not the same. In this case, we set the cluster sizes to be arranged in a proportional sequence with a ratio of 0.8, that is, “imbalanced”. In addition, we can control the dropout probability of two datasets by adjusting the “dropout.mid” parameter (“dropout.mid” = −1, −0.5, 0 and 0.5, “de.facScale” = 0.2, “dropout.shape” = −1). In response to the various possible parameter combinations above (16 cases in total), we generated ten datasets by setting different random seeds, and we used the median value of ten results for evaluation. When the whole source and target datasets were used in the experiment, we first compared the average ARI and annotation accuracy in 16 cases. From Table 2, we have scmap (ARI 0.6488, annotation accuracy 0.8265), scANVI (ARI 0.2765, annotation accuracy 0.5784), ItClust (ARI 0.8895, annotation accuracy 0.9477), and scAnCluster achieved the best performance (ARI 0.9525, annotation accuracy 0.9780). Our method outperforms the other three algorithms in almost every scenario (see Figure 2). In particular, when other parameters are fixed, and the dropout probability increases, the ARI and annotation accuracy of each method show a decreasing trend, but scAnCluster is always located at the top of the four lines (see Supplementary Figure S1). When parameter dropout.mid changes from −0.5 to 0 and 0.5, it is worth noting that scmap and ItClust decline significantly, while the decline trend of scAnCluster is relatively flat, essentially because we have built in the probability of zero-inflation in the model.

**Figure 3:**
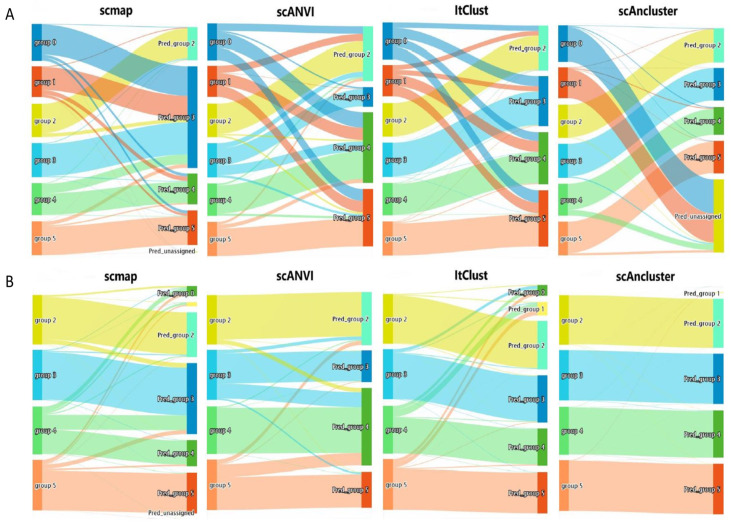
Simulation dataset analysis. (**A**) Sankey plots for four methods on one mixed dataset that removes group 0 and group 1 in the source dataset. (**B**) Sankey plots for four methods on one mixed dataset that removes group 0 and group 1 in the target dataset.

We performed two more groups of control experiments by deleting cell types labeled 0 and 1 from the source and target datasets, respectively. When we removed them in the source dataset, the number of cell types provided for reference decreased, and we found that scAnCluster performed best in ARI, while ItClust performed best in terms of annotation accuracy (see Table 2, Supplementary Figures S2 and S3). Actually, ItClust could assign all cells in the target dataset to known cell types in the source dataset. Our method, however, tended to divide all cells into *k* clusters according to the predetermined total number of clusters. We used one mixed dataset under “equal”, “balanced” and “dropout.mid” 0.5 setting as an illustration, effectively removing label 0 and 1 in the source dataset in advance. From the Sankey plot (see Figure 3A), we can see that ItClust distributes those cells with label 0 and label 1 in the target dataset into four other labels. Nearly the same thing happens with scmap and scANVI. Surprisingly, scAnCluster assigned more than 90% of those cells to the "unassigned" label and further clustered them into two disjoint groups. This is mainly due to self-supervised similarity fusion and reinforcement learning strategy. These two novel unknown categories are dissimilar to the four known cell types, and thus far away from the area where they are located. At the same time, due to their high internal similarity, they have gradually formed two new groups. Therefore, it reflects the superiority of our method in discovering novel cell types. Interestingly, when we removed these two clusters in the target dataset, whether it was ARI or annotation accuracy, scAnCluster proved to be far superior to scmap, scANVI and ItClust (see Table 2, Supplementary Figures S4 and S5). We also demonstrate the annotation results of one mixed dataset that first removes two groups in the target dataset by Sankey graph (see Figure 3B). Compared to the other three methods, the clustering and annotation results of scAnCluster are more concentrated and not so scattered. It rarely projects cells in the target dataset onto inaccurate cell types. Given a complete and pure reference standard, the supervised training and self-supervised training in scAnCluster can help match cells in the target dataset to the most similar cell population in the source dataset. However, scmap has a tendency to randomly assign cells in the target dataset to cell types in the source dataset, owing to the subjective threshold selection. ItClust tends to assign all cells in the target dataset to all known cell types, ignoring the possibility that partial cell types only exist in the source dataset or in the target dataset. Additional sankey plots can be seen in Supplementary Figure S6 and Figure S7A. We found the performance of scANVI to be relatively sluggish throughout the simulation experiment, showing that it failed to learn the low-dimensional manifold where the data is located.

### 3.3. Inter-Dataset Clustering Analysis

**Figure 4:**
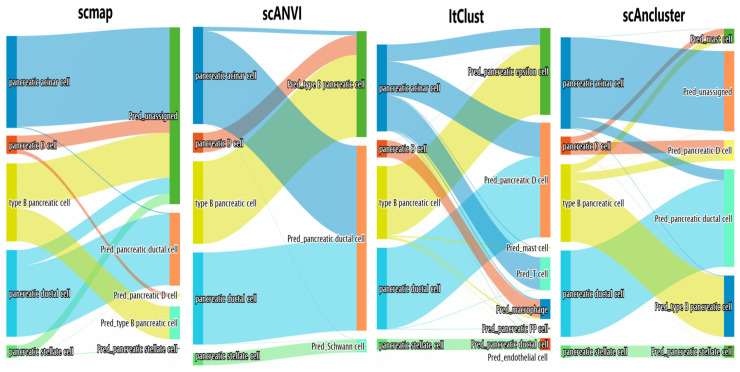
Inter-dataset analysis. Sankey plots for four methods on the incomplete “Baron_human+Enge” dataset that removes the pancreatic acinar cells in the “Baron_human” dataset.

We selected two groups of datasets from pancreas tissue and retinal tissue: “Baron_human” [36], “Enge” [37], “Muraro” [38], “Segerstolpe” [39], “Xin” [40] and “Macosko” [41], “Shekhar” [42]. Their sequencing platform, cell number, gene number and cell type number can be seen in Supplementary Table S2. For the pancreas group, we first assume that “Baron_human” is the source dataset and that “Enge” is the target dataset. The former contains all cell types of the latter. In order to explore the effect of mixing multiple datasets on the results, we combined five pancreas datasets listed above for clustering and annotation analysis, where “Baron_human” is the source dataset and the other four together form the target dataset. This mixed target dataset has ten cell types that are contained in the source dataset. For the retina group, we assume that “Macosko” is the source dataset and that “Shekhar” is the target dataset. Five cell types are shared in common between the two datasets. Our experiments were mainly divided into two parts. The first part used all cells in the source dataset and target dataset for clustering and annotation analysis. The second part eliminated the first two largest cell types in common from the source dataset. From the results of the pancreas group in the first part of Table 3, it can be seen that scAnCluster always gives the highest ARI and annotation accuracy under the three experimental settings. scmap performs poorly most of the time. This performance illustrates how merely calculating the correlation between cluster centers of known cell types and the cells of unknown cell types ignores the global structure of the entire cell clusters in the source dataset. Although the annotation accuracy of scANVI is comparable to that of scAnCluster, its ARI value showed a clear cliff-like decline when we removed the major common cell types from the source dataset, indicating that scANVI did not perform as well as scAnCluster with aspect to the discovery of novel cell types. A similar phenomenon also appeared in ItClust, and when the cell types in the source dataset were not removed, the annotation accuracy of ItClust was almost zero, implying that ItClust did not correctly utilize the label information in the source dataset, but simply finished clustering on the target dataset. We also used the Sankey plot to support our claim (see Supplementary Figure S7B). When all cells participate in clustering, ItClust annotates most cell types in the target dataset into another cell type. Meanwhile, scmap annotates massive pancreatic stellate cells and type B pancreatic cells as “unassigned”. Similarly, scANVI only succeeds in annotating less than 5% of pancreatic D cells. In contrast, scAnCluster, maps most cells in each cell type to the correct type successfully. When we deleted the pancreatic acinar cells in the source dataset (see Figure 4), only scAnCluster and scmap annotated more than 85% of this cell type as “unassigned”, while the other two methods projected them to another incorrect type. For the experiment of five mixed datasets, we can get a similar conclusion (see second part of Table 3 and Supplementary Figure S8). Regardless of whether the source dataset is complete or incomplete, scAnCluster always performs best, which fully shows that our method has excellent robustness to integrate multiple datasets sequenced with different sequencing machines and protocols. The results of the retina group are shown in the third part of Table 3 and Supplementary Figure S9A. We can see that ItClust and scmap perform unsatisfactorily in all three settings. By removing large cell types available for reference, scANVI exhibits different degrees of collapse in its clustering and annotation performance. On the other hand, even if the common large cell types in the source dataset are removed, the clustering effect of scAnCluster is still excellent on the target dataset, and the annotation accuracy is also very high on the cell types that can be annotated, fully reflecting the superiority and robustness of scAnCluster in meeting these challenging annotation tasks. In general, our method outperforms the three other supervised clustering algorithms in inter-dataset clustering analysis.

### 3.4. Intra-Dataset Clustering Analysis

**Figure 5:**
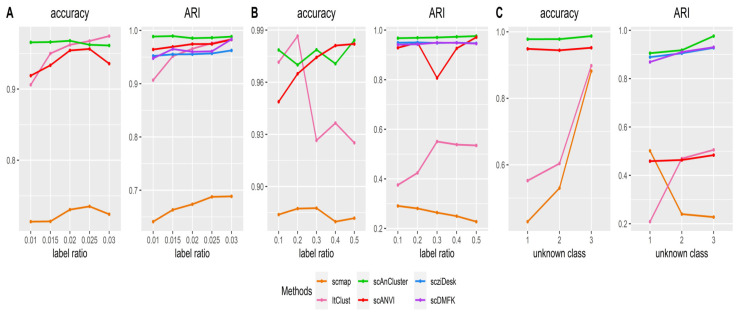
Intra-dataset analysis. (**A**–**C**) Line plots of ARI and annotation accuracy values for four methods in the “random”, “small(r)” and “large(c)” experiments on the "Campbell" dataset, respectively.

Actually, scAnCluster can also be applied to supervised clustering and annotation on an intra-dataset. Given the low sensitivity of scRNA-seq assays, some cells may be easier to cluster and annotate than others. In this case, a single dataset can allow partial cells to be well annotated in advance. We can use these well-labeled cells to help and guide the clustering and annotation process of the entire dataset. In this regard, we selected a dataset from brain tissue, “Campbell” [43], which is mainly divided into eight categories, and the neurons account for the majority. We also treat scDMFK and scziDesk as methods for comparing clustering performance, although their annotation accuracy cannot be calculated. We designed three groups of experiments to validate the effectiveness of each method in terms of clustering and self-projection annotation. The first group, namely “random”, allows the labels of partial cells to be known randomly according to a certain ratio (0.01, 0.015, 0.02, 0.025 and 0.03). These cells belong to the source dataset, and the remaining cells are included in the target dataset. The second group, namely “small(r)”, extracts only part of the cells in the five small cell types in order of increasing probability (0.1, 0.2, 0.3, 0.4 and 0.5) into the source dataset. The remaining cells are classified as the target dataset. The third group, namely “large(c)”, automatically divides the first three large cell types into the target dataset in order of increasing number. For other remaining cell types, each cell is included in the source dataset with a probability of 0.5. These procedures are repeated five times, and we take their median ARI and annotation accuracy values for evaluation (see Table 4). In the “random” experiment (see Figure 5A), all cell types are set to known. Even if only one to three percent of the labels can be used as guidance for clustering, the ARI and annotation accuracy of scAnCluster are still above 0.96, outperforming scmap, scANVI and ItClust. This kind of superiority is more obvious when the other two groups are similarly challenged with clustering and annotation tasks (see Figure 5B,C). It can also be seen that under the supervision of a small number of labels, the ARI value of scAnCluster is about three percentage points higher than that of scDMFK and scziDesk. At the same time, scAnCluster can also annotate unlabeled cells well, which is impossible for scDMFK and scziDesk. When we divide the largest cell type, "neuron", into the target dataset, Sankey plots (see Supplementary Figure S9B) show that both scANVI and ItClust assign them to other cell types like “oligodendrocyte”, while scmap and scAnCluster project more than 95% of neurons to “unassigned”. However, scmap gives the unsatisfactory annotation results on other cell types, leading to low annotation accuracy and ARI values. These phenomena fully show that the performance of scmap, scANVI and ItClust is highly dependent on the quality and quantity of well-labeled data. Certainly, it also reflects that scAnCluster can better integrate known label information and is more suitable than the others for discovering novel cell types.

## 4. Discussion and Conclusions

Single-cell transcriptome sequencing technologies have brought unprecedented capabilities to the study of tissue heterogeneity. Two essential analytical procedures are cell clustering and annotation, and they have always been very challenging and labor-intensive. In this paper, based on scDMFK and scziDesk, we use the available cell label information to develop a novel deep learning model that integrates single-cell clustering and annotation. Our method, scAnCluster, has the following highlights. Firstly, our model can perform both cell clustering and annotation for intra-datasets, as well as for inter-datasets. Secondly, our model shows strong discriminatory power in discovering novel cell types that are absent in the reference data. Thirdly, our model is end-to-end so that it does not require intensive feature engineering. It incorporates some mainstream deep learning techniques, including supervised learning, self-supervised learning and unsupervised learning. In order to better demonstrate the robustness and reliability of our method, we also implemented several groups of control experiments.

### 4.1. Influence of Highly Variable Genes Number

**Figure 6:**
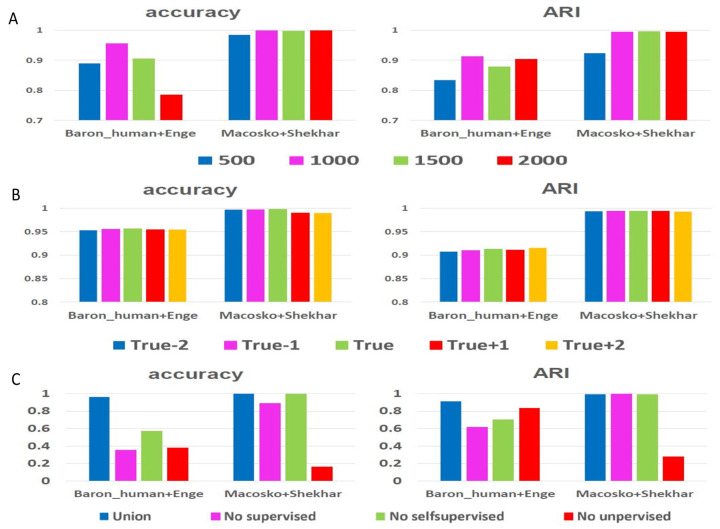
Ablation study on the inter-datasets. (**A**–**C**) Histograms of ARI and annotation accuracy values for scAnCluster in three groups of case control experiments on two inter-datasets, respectively.

Single-cell RNA-seq data is usually highly noisy. Consequently, selecting some informative, highly variable genes for clustering analysis helps to improve the clustering performance. During the experiments in the text, we selected 1000 highly variable genes by default. To illustrate the effect of highly variable genes on the results, we conducted control experiments on two groups of inter-datasets. The number of highly variable genes changed from 500 and 1000 to 1500 and 2000. From the results in Figure 6A, we find that the ARI value of 500 genes on the “Macosko+Shekhar” dataset is significantly lower than that of the other three conditions, while the difference of annotation accuracy is not evident. For “Baron_human+Enge”, a similar phenomenon occurs with 500 genes in terms of ARI value, but the selection of 1000 genes is unique for annotation accuracy. Therefore, considering the calculation time and memory limitations, we selected 1000 highly variable genes by default for clustering analysis. Certainly, for complex datasets with massive cell types, we also recommend that users select more highly variable genes for clustering analysis.

### 4.2. Influence of Total Cluster Number

Inferring the number of categories in the dataset has always been a challenging problem for statisticians and machine learning researchers. However, in single-cell clustering analysis, it seems that confusion over how to choose cell type number is essentially settled since this decision depends on the investigator’s concerns and aims. In previous experiments, we took the total cell type number by default as the true one of the whole mixed dataset and recorded it as *k*. To explore the impact of the total number of cell types on the ARI and annotation accuracy, we let the total cell type number to be selected from {k−2,k−1,k,k+1,k+2} and implemented experiments on two groups of inter-datasets. From the results in Figure 6B, the changes in ARI and annotation accuracy values are relatively small, fully demonstrating the robustness of scAnCluster to total cell type number. Therefore, we recommend that users employ a rough estimation of the cell type number on the mixed dataset before applying our method.

### 4.3. Importance of Three Parts of Learning Strategies

**Figure 7:**
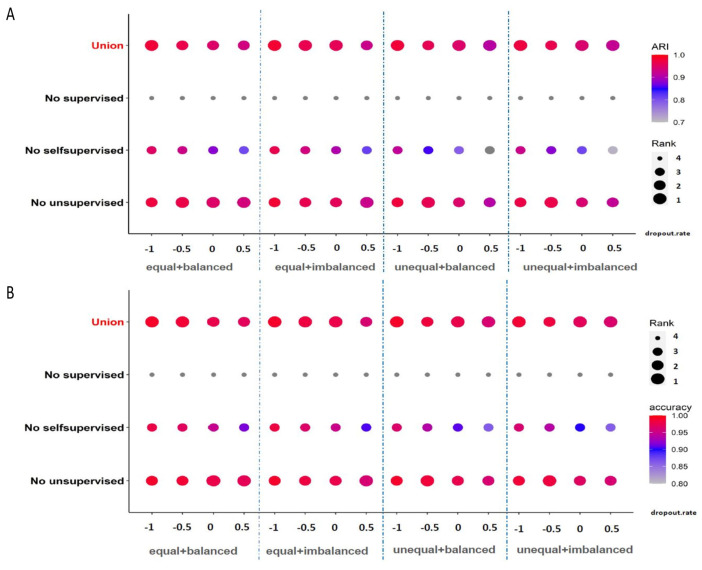
Ablation study on the simulation datasets. (**A**,**B**) Bubble plots of ARI and annotation accuracy values for scAnCluster in four groups of combining learning strategies, respectively.

**Figure 8:**
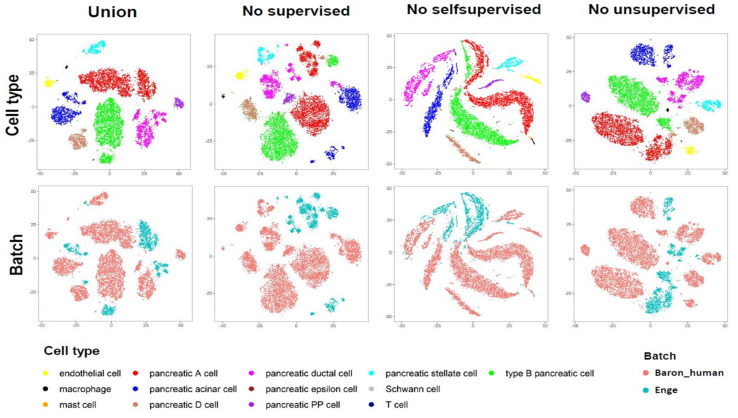
Visualization plots in 2D plane for scAnCluster in four groups of combining learning strategies on the “Baron_human+Enge” dataset.

So far, we have not discussed the contribution and significance of supervised learning, self-supervised learning and unsupervised learning in our model because in previous experiments, we always combined them together for training. In order to show the indispensability of these three parts of the learning process, we consider the method of controlling variables, as a kind of loss/gain-of-function exercise, by removing one of the three parts and retaining the other two parts for training. From the results on simulation datasets, when we remove supervised learning, which is classification learning on the source dataset, the ARI and annotation accuracy values seriously collapse (see Figure 7). Similarly, when we remove the self-supervised similarity fusion training on the mixed datasets, these two evaluation indicators also show a significant decline. In the real data of “Macosko” and “Shekhar”, we can see that removing unsupervised soft clustering training will make clustering and annotations less ideal (see Figure 6C). For “Baron_human” and “Enge”, throwing away any of the three parts will lead to unsatisfactory results (see Figure 6C). Further, we also show the results of these comparative experiments on the “Baron_human + Enge” dataset with a two-dimensional visualization in Figure 8. Specifically, we use UMAP (Uniform Manifold Approximation and Projection) [44] to reduce the latent space representation obtained in each case to a two-dimensional plane. In order to distinguish these two datasets, we have also produced corresponding visualizations of the batch index. We focused on the four main cell types common to both datasets, namely “type B pancreatic cell”, “pancreatic A cell”, “pancreatic ductal cell” and “pancreatic acinar cell”. When combining the three learning techniques, these four cell types in the “Enge” dataset are well mapped near the corresponding cell types in the “Baron_human” dataset. However, removing supervised classification learning will result in improper annotation of “type B pancreatic cell”, “pancreatic A cell” and “pancreatic ductal cell”. A similar phenomenon also occurred when self-supervised similarity learning was removed. When eliminating unsupervised soft clustering learning, type B pancreatic cells and pancreatic A cells in the “Enge” dataset could not be well separated, which affected cell type annotations. Overall, the three parts of our model are complementary. Supervised classification learning affords the latent space needed to obtain basic discriminative ability, thus guaranteeing the smooth progress of annotation. The use of self-supervised learning for bootstrapping the cell latent representation trades off the representation quality with its generality for discovering novel cell types. Jointly carrying out unsupervised clustering on both labeled reference data and unlabeled target data can reinforce the annotation task, while avoiding forgetting. When they function together, they can facilitate cell clustering and annotation more accurately and convincingly.

Based on previous discussion, our method, scAnCluster, whether in simulation data or real data analysis, has shown competitive performance compared to that of each existing single-cell RNA-seq supervised clustering algorithm tested. In the simulation experiments, our method is robust to various settings of dataset size, cluster size and dropout probability. On intra-datasets and inter-datasets, our method shows a strong ability to explore and discover new cell types with the only partial overlap in cell type compositions. Such evidence ensures that our method will stand out among existing single-cell clustering and annotation algorithms. The combination experiments of multiple datasets from the same tissue also validate the stability and robustness of our method for integrating data generated by different sequencing technologies. In addition, although scAnCluster is not as fast as scmap, it still has an advantage over ItClust and scANVI in calculation efficiency (see Supplementary Table S3). Considering the running speed, clustering and annotation performance, scAnCluster is an ideal choice for users. With the rise and maturity of single-cell multiomics sequencing technologies, we can recommend applying our algorithm ideas to the design of integrated multiomics analysis methods. Of course, our method also has some potential faults. For example, the strong batch effect between source dataset and target dataset may not be completely eliminated by simply concatenating the gene expression data with the batch ID. Therefore, we suggest that users take advantage of other scRNA-seq customized batch correction softwares, such as pagoda2 [45] (see Supplementary Table S4), before using our method.

## Figures and Tables

**Table 1:** Single-cell RNA-seq supervised annotation tools named in this study.

Tools	Underlying Algorithm	Requiring Annotated Genes	“Unassigned” Function
CellAssign	Bayes probabilistic model	Yes	No
Garnett	Generalized linear model	Yes	Yes
SingleR	Spearman correlation to training set	No	No
Moana	SVM with linear kernel	Yes	No
scmap(cluster)	Nearest median classifier	No	Yes
scANVI	Semi-supervised deep generative model	No	Yes
ItClust	Deep transfer learning model	No	Yes
scAnCluster	Integration of deep supervised, self-supervised, unsupervised learning	No	Yes

**Table 2:** Average performance of scmap, scANVI, ItClust and scAnCluster in 16 cases for different simulation settings. “Whole” refers to the experiments with complete data. “source(1)” and “source(2)” refer to the deletion of cell types with label {0} and label {0, 1} in the source dataset, respectively. Similarly, “target(1)” and “target(2)” refer to the deletion of cell types with label {0} and label {0, 1} in the target dataset, respectively.

	ARI				
	whole	source(1)	source(2)	target(1)	target(2)
scmap	0.6488	0.4635	0.3007	0.6605	0.6529
scANVI	0.2765	0.1946	0.1365	0.2798	0.3280
ItClust	0.8895	0.6107	0.3773	0.8416	0.7798
scAnCluster	0.9525	0.8039	0.5425	0.9453	0.9347
	**Annotation Accuracy**				
	**whole**	**source(1)**	**source(2)**	**target(1)**	**target(2)**
scmap	0.8265	0.8377	0.8626	0.8216	0.8111
scANVI	0.5784	0.5953	0.6547	0.5960	0.6398
ItClust	0.9477	0.9539	0.9611	0.9021	0.8509
scAnCluster	0.9780	0.9078	0.8637	0.9748	0.9704

**Table 3:** Performance of scmap, scANVI, ItClust and scAnCluster on three groups of inter-datasets, that is, “Baron_human”(source) and “Enge”(target), “Baron_human”(source), “Enge”(target), “Muraro”(target), “Segerstolpe”(target) and “Xin”(target), “Macosko”(source) and “Shekhar”(target). “whole” refers to the experiments with complete data. “large(1)” and “large(2)” refer to the deletion of overlapping cell types with the largest and first two largest sizes from the source dataset, respectively.

	Baron_human+Enge					
	ARI			Annotation Accuracy		
	whole	large(1)	large(2)	whole	large(1)	large(2)
scmap	0.4840	0.4864	0.2727	0.6621	0.6456	0.5556
scANVI	0.8923	0.6028	0.4949	0.9404	0.9206	0.8431
ItClust	0.8542	0.4930	0.3255	0.0184	0.0117	0.0000
scAnCluster	0.9088	0.8722	0.8363	0.9588	0.9252	0.8660
	**Baron_human+Enge+Muraro+Segerstolpe+Xin**					
	**ARI**			**Annotation Accuracy**		
	**whole**	**large(1)**	**large(2)**	**whole**	**large(1)**	**large(2)**
scmap	0.6772	0.4454	0.3630	0.7819	0.7536	0.7345
scANVI	0.8804	0.7585	0.6866	0.8926	0.8413	0.8199
ItClust	0.8808	0.4167	0.3553	0.0236	0.0132	0.0005
scAnCluster	0.8918	0.8086	0.7730	0.9303	0.8845	0.8646
	**Macosko+Shekhar**					
	**ARI**			**Annotation Accuracy**		
	**whole**	**large(1)**	**large(2)**	**whole**	**large(1)**	**large(2)**
scmap	0.4264	0.4123	0.0167	0.1182	0.3543	0.2583
scANVI	0.9961	0.8024	0.2800	0.8895	0.9961	0.7647
ItClust	0.2349	0.4949	0.4240	0.5922	0.9484	0.6215
scAnCluster	0.9923	0.9920	0.9719	0.9984	0.9987	0.9989

**Table 4:** Average performance of scmap, scANVI, ItClust, scAnCluster, scDMFK and scziDesk on the “Campbell” dataset. “random” refers to randomly selecting a certain percentage of cells to form a source dataset. “small(r)” refers to dividing the cells in five small clusters into the source dataset according to a certain proportion. “large(c)” means that the cells in some large clusters are automatically classified as the target dataset, that is, they do not appear in the source dataset.

	ARI			Annotation Accuracy		
	random	small(r)	large(c)	random	small(r)	large(c)
scmap	0.6708	0.2625	0.3230	0.7234	0.8841	0.6137
scANVI	0.9731	0.9166	0.4688	0.9398	0.9493	0.9584
ItClust	0.9567	0.4847	0.3947	0.9521	0.9764	0.6849
scAnCluster	0.9876	0.9713	0.9332	0.9647	0.9764	0.9810
scDMFK	0.9634	0.9457	0.9031			
scziDesk	0.9564	0.9489	0.9073			

## Supplementary Materials

The following are available at https://www.mdpi.com/2073-4425/11/7/792/s1, Table S1: Weight hyperparameters setting in various experiments; Table S2: The information for all real datasets from different organs; Table S3: Average running time for each tested tools in various experiments; Table S4: Performance of scAnCluster with or without the preprocessing by pagoda2 on “Baron_human+Enge+Muraro+Segerstolpe+Xin” dataset. "whole" refers to the experiments with complete data. "large(1)" and "large(2)" refer to the deletion of overlapping cell types with the largest and first two largest sizes from the source dataset, respectively; Figure S1: Simulation dataset analysis. (A and B) Change of ARI and annotation accuracy values with the increasing dropout rate for four methods in four groups of simulation experiments using the whole mixed datasets, respectively; Figure S2: Simulation dataset analysis. (A and B) Change of ARI and annotation accuracy values with the increasing dropout rate for four methods in four groups of simulation experiments using the incomplete mixed datasets that remove the group 0 in the source datasets, respectively; Figure S3: Simulation dataset analysis. (A and B) Change of ARI and annotation accuracy values with the increasing dropout rate for four methods in four groups of simulation experiments using the incomplete mixed datasets that remove the group 0 and group 1 in the source datasets, respectively; Figure S4: Simulation dataset analysis. (A and B) Change of ARI and annotation accuracy values with the increasing dropout rate for four methods in four groups of simulation experiments using the incomplete mixed datasets that remove the group 0 in the target datasets, respectively; Figure S5: Simulation dataset analysis. (A and B) Change of ARI and annotation accuracy values with the increasing dropout rate for four methods in four groups of simulation experiments using the incomplete mixed datasets that remove the group 0 and group 1 in the target datasets, respectively; Figure S6: Simulation dataset analysis. (A) Sankey plots for four methods on one mixed complete dataset. (B) Sankey plots for four methods on one mixed dataset that removes group 0 in the source dataset; Figure S7: Simulation and real dataset analysis. (A) Sankey plots for four methods on one mixed dataset that removes group 0 in the target dataset. (B) Sankey plots for four methods on the whole “Baron_human+Enge” dataset; Figure S8: Real dataset analysis. Sankey plots for four methods on the whole “Baron_human+Enge+Muraro+Segerstolpe+Xin” dataset. The upper row represents ItClust and scAnCluster, and the lower row represents scANVI and scmap; Figure S9: Real dataset analysis. (A) Sankey plots for four methods on the whole “Macosko+Shekhar” dataset. (B) Sankey plots for four methods in the “large(c)” experiment on the “Campbell” dataset that automatically divides all neurons into the target dataset.
