# Supplementary material for: Integrating Deep Supervised, Self-Supervised and Unsupervised Learning for Single-Cell RNA-seq Clustering and Annotation
Source: Genes (Basel). 2020 Jul 14;11(7):792. doi: 10.3390/genes11070792 (PMC7397036; doi:10.3390/genes11070792)
Supplement: Supplementary file 1 [file genes-11-00792-s001.pdf]

Supplementary for scAnCluster

Liang Chen[1,], Yuyao Zhai[2], Qiuyan He[1,], Weinan Wang[1,], Minghua Deng[1,3,4,]

[1]School of Mathematical Sciences, Peking University, Beijing, China 100871;
[2]Mathematical and Statistical institute, Northeast Normal University, Changchun, China 130024;
[3]Center for Quantitative Biology, Peking University, Beijing, China 100871;
[4]Center for Statistical Science, Peking University, Beijing, China, 100871;
clandzyy@pku.edu.cn(Chen L)
zhaiyy375@nenu.edu.cn(Zhai Y)
heqy@pku.edu.cn(He Q)
wangweinan@pku.edu.cn(Wang W)
dengmh@pku.edu.cn(Deng M)

1 Details of using other tools.

scmap(cluster): We used scmap provided in github: https://github.com/hemberg-lab/scmap/blob/master/vignettes/scmap.Rmd. As the package designed, the input form of scmap must be "SingleCellExperiment" class. Then we first select the most informative features (genes) from our input source dataset. After that, we calculate the median gene expression for each cluster and save the "scmap-cluster" index of the source dataset. Once the "scmap-cluster" index has been generated, we can use it to project our target dataset to itself. Finally we can obtain the results of cell label assignements for target dataset.
scANVI: We used scANVI provided in github: https://github.com/chenlingantelope /HarmonizationSCANVI. As the codes suggested, we first transform the mixed dataset into anndata form and record the index of the known cell type label. Then we use the functions "scvi.models.SCANVI" and "scvi.inference.annotation.Custom SemiSupervisedTrainer" to construct and train the deep generative mixture model. Finally we can the class posterior probabilities of each cell in target dataset. Naturally, we can assign each cell to the cell type with the highest posterior probability. As for the training epochs, we follow the authors' suggestion and set it as 400 for dataset with less than 10000 cells and 200 for dataset with less than 100000 cells.
ItClust: We used ItClust provided in github: https://github.com/jianhuupenn /ItClust/blob/master/tutorial/tutorial.ipynb. As the author suggested, we first construct the source anndata object and target anndata object. Then we use its "transfer_learning_clf" function to build the transfer learning framework and take the source data and target data as inputs. ItClust includes preprocessing steps automatically, that is, filtering of cells/genes, normalization, scaling and selection of highly variables genes. Selection of top highly variable genes is based on the target dataset only. ItClust would first pretrain a stack autoencoder network on the source data and then take the well-trained network parameter and

cluster centers as the initial values of target network. Finally we use the "predict" function to obtain the cell type assignment of each target cell.

2 Parameter setting

λ_1 and λ_2 are two weight hypeparameters that control training balance between different tasks. The following table gives their experimental setting in simulation datasets and real datasets.

Table 1: Weight hyperparameters setting in various experiments.

	default setting	
experiments	λ_1	λ_2
simulation	0.01	0.01
Baron_human+Enge	0.01	0.1
Baron_human+Enge+Muraro+Segerstolpe+Xin	0.01	0.01
Macosko+Shekhar	0.1	0.1
Campbell	0.1	0.001

3 Real data sets information

In the real data analysis section, we select eight real datasets that have made the purified cell types available to public. We summarize their basic information into following table.

Table 2: The information for all real datasets from different organs.

	real data information					
dataset	organ	platform	cell ontology	cell	gene	zero percent
Baron_human	Pancreas	inDrop	13	8569	20125	90.62%
Enge	Pancreas	Smart-seq2	6	2282	23368	86.05%
Muraro	Pancreas	CEL-Seq2	9	2122	19046	73.02%
Segerstolpe	Pancreas	Smart-seq2	10	1070	25453	77.97%
Xin	Pancreas	SMARTer	4	609	39851	85.12%
Macosko	Retinal	Drop-seq	12	44808	23288	96.95%
Shekhar	Retinal	Drop-seq	5	26830	13166	93.34%
Campbell	Brain	Drop-seq	8	20921	26754	92.79%

4 Running time of each tool

Throughout the experiments, each tool was calculated on the same machine. In the table below, we provide the running time of each tool in every experiment, which is the average value of ten results. Each experiment was conducted on complete source dataset and target dataset. It can be seen that scmap is the fastest method, but its clustering and annotation performance is not very good. scAnCluster runs faster than ItClust and scANVI, and its results are better.

The running speed of ItClust mainly depends on the size of the source dataset. For example, when performing self-projection intra-dataset analysis, the source dataset is smaller, and ItClust's speed is faster than scAnCluster, because scAnCluster needs to pre-train the entire single dataset.

Table 3: Average running time for each tested tools in various experiments.

experiments	tested tools and time (min)			
	ItClust	scAnCluster	scANVI	scmap
simulation	4.23	2.87	21.24	< 1
Baron_human+Enge	7.57	7.70	73.54	< 1
Baron_human+Enge+Muraro+Segerstolpe+Xin	10.20	7.40	93.65	1.16
Macosko+Shekhar	41.48	29.00	271.75	3.88
Campbell	3.11	13.29	107.07	1.50

5 Apply pagoda2 for preprocessing

In the text, we have pointed out that when the mixed dataset has a strong batch effect, we recommend using pagoda2 for data preprocessing, before applying our method. We take the "Baron_human+Enge+Muraro+Segerstolpe+Xin" dataset as an example. Similarly, we utilize the top 1000 highly variable genes of the dataset processed by pagoda2 for the experiment. The results are shown in the following table. We can see that after using pagoda2 for preprocessing, the ARI and annotation accuracy has improved significantly in almost all cases.

Table 4: Performance of scAnCluster with or without the preprocessing by pagoda2 on "Baron_human+Enge+Muraro+Segerstolpe+Xin" dataset. "whole" refers to the experiments with complete data. "large(1)" and "large(2)" refer to the deletion of overlapping cell types with the largest and first two largest sizes from the source dataset, respectively.

	ARI			Annotation accuracy		
	whole	large(1)	large(2)	whole	large(1)	large(2)
scAnCluster	0.8918	0.8086	0.7730	0.9303	0.8845	0.8646
scAnCluster+Pagoda2	0.9281	0.8343	0.8075	0.9531	0.8880	0.9042

6 Additional figures

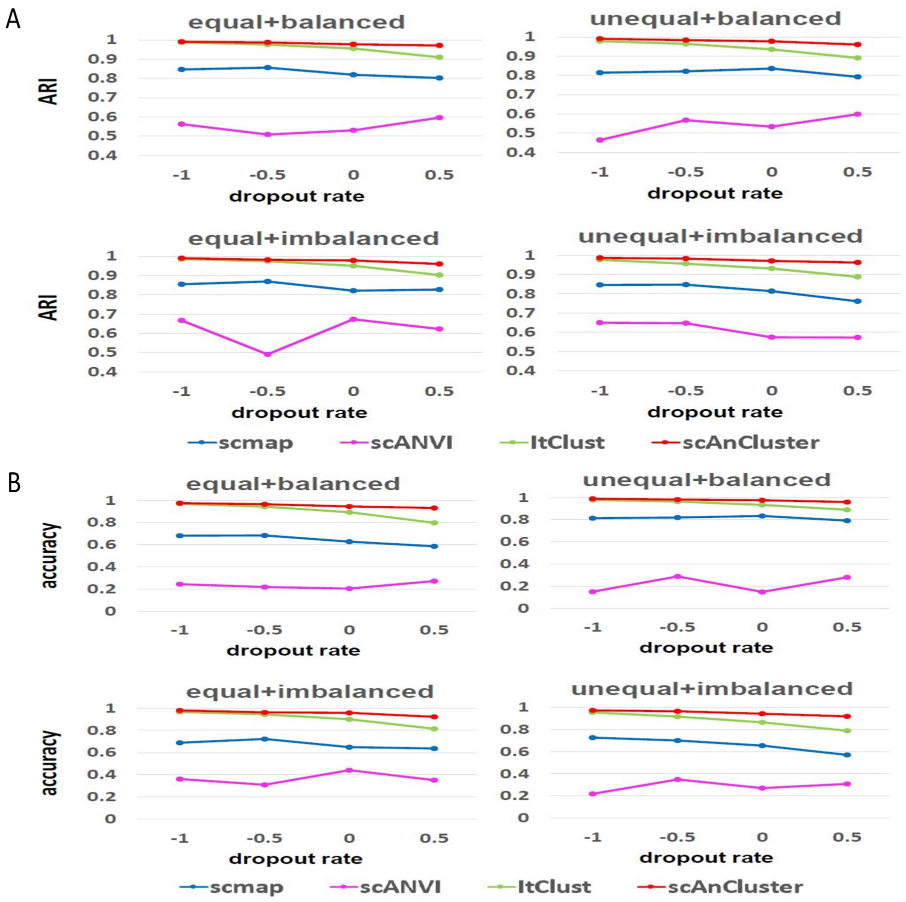

Fig. 1: Simulation dataset analysis.(A and B)Change of ARI and annotation accuracy values with the increasing dropout rate for four methods in four groups of simulation experiments using the whole mixed datasets, respectively.

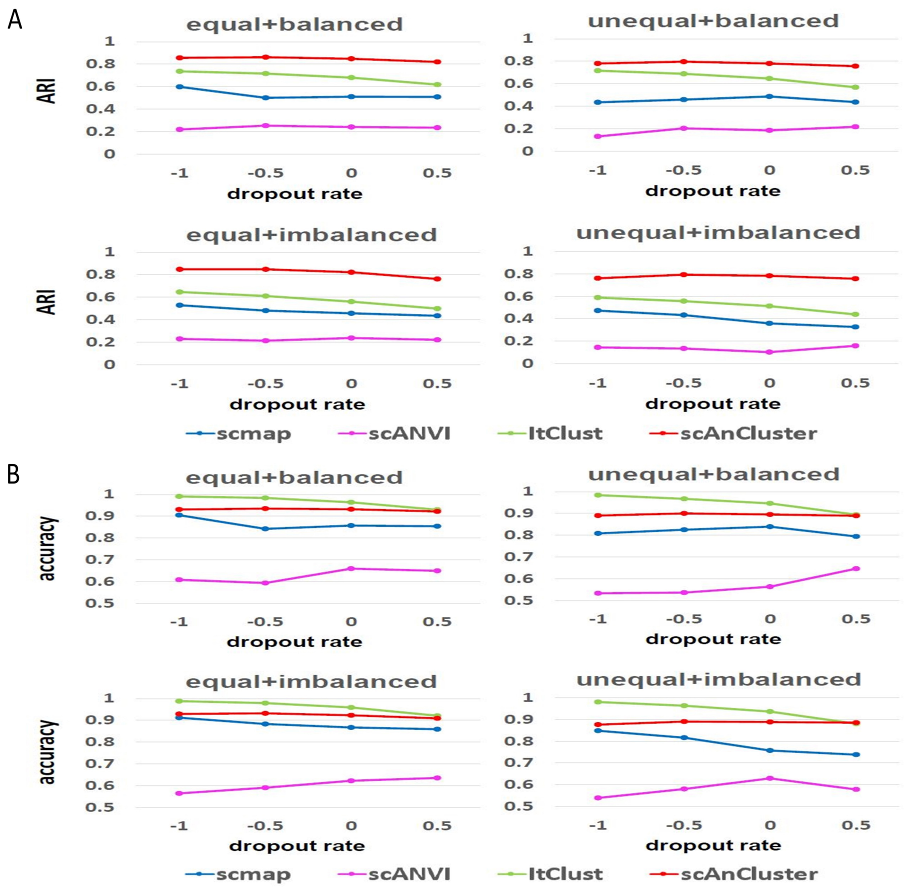

Fig. 2: Simulation dataset analysis.(A and B)Change of ARI and annotation accuracy values with the increasing dropout rate for four methods in four groups of simulation experiments using the incomplete mixed datasets that remove the group 0 in the source datasets, respectively.

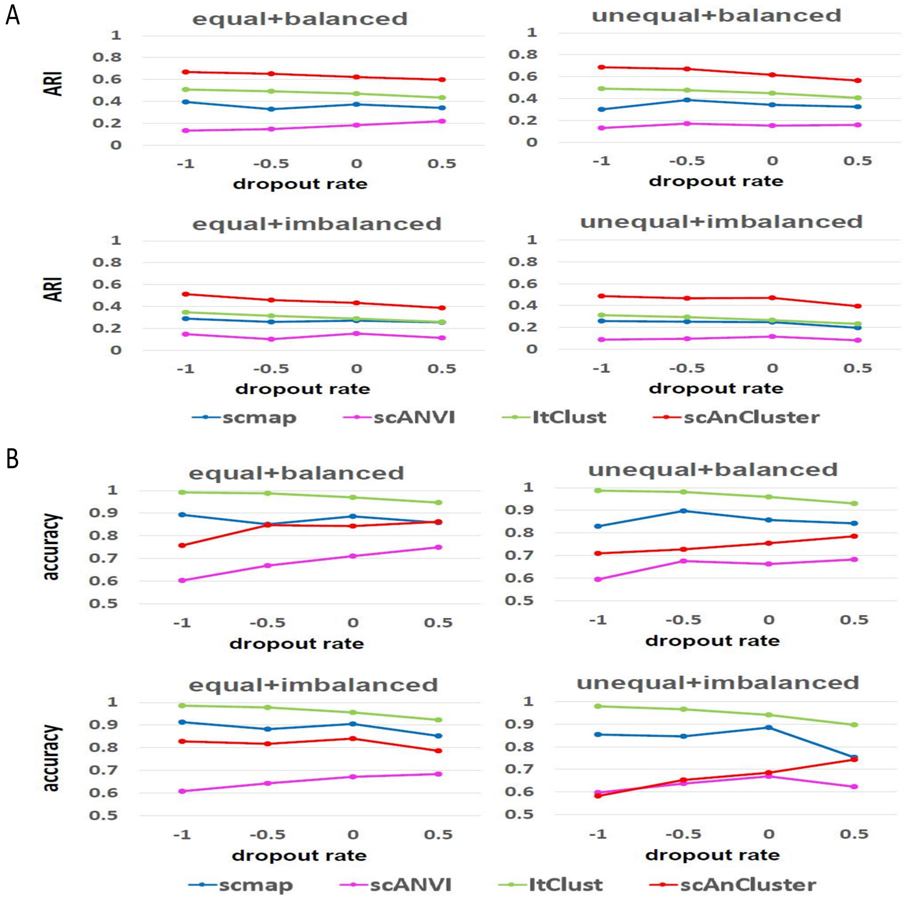

Fig. 3: Simulation dataset analysis.(A and B)Change of ARI and annotation accuracy values with the increasing dropout rate for four methods in four groups of simulation experiments using the incomplete mixed datasets that remove the group 0 and group 1 in the source datasets, respectively.

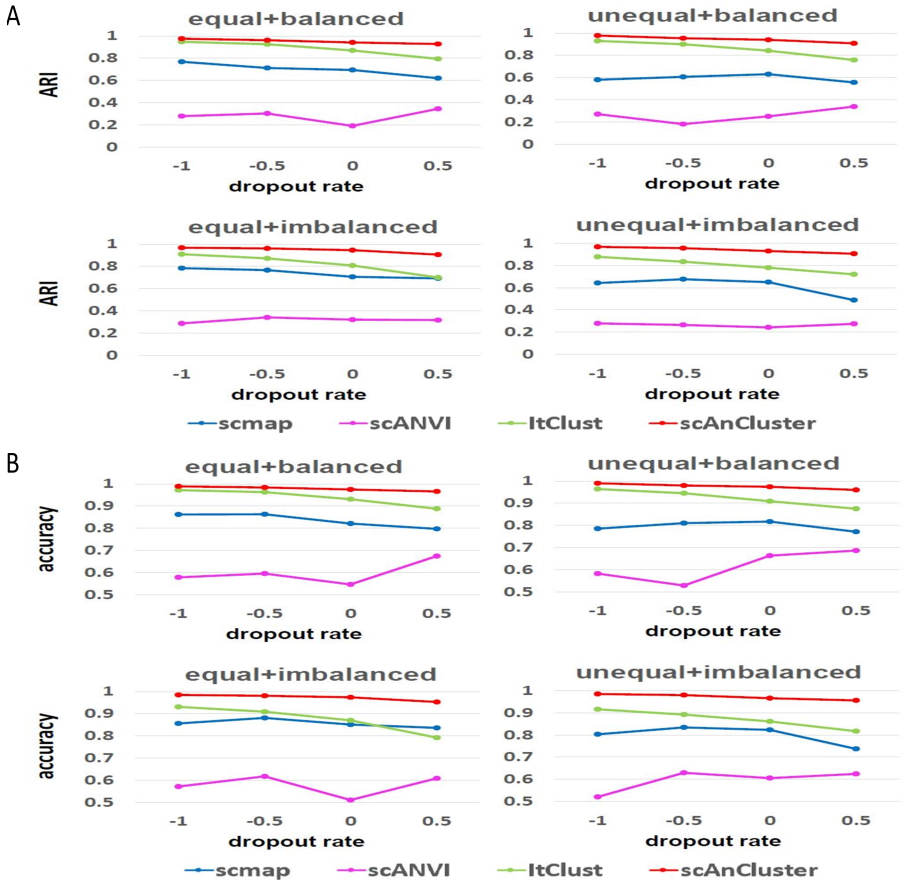

Fig. 4: Simulation dataset analysis.(A and B)Change of ARI and annotation accuracy values with the increasing dropout rate for four methods in four groups of simulation experiments using the incomplete mixed datasets that remove the group 0 in the target datasets, respectively.

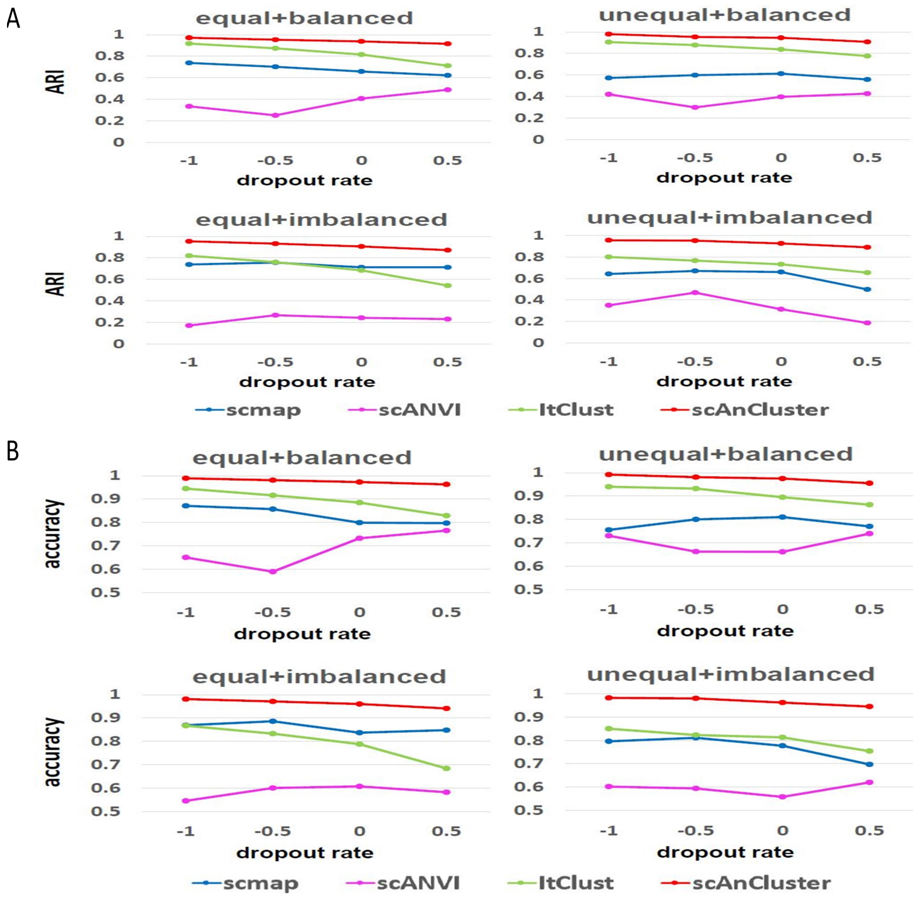

Fig. 5: Simulation dataset analysis.(A and B)Change of ARI and annotation accuracy values with the increasing dropout rate for four methods in four groups of simulation experiments using the incomplete mixed datasets that remove the group 0 and group 1 in the target datasets, respectively.

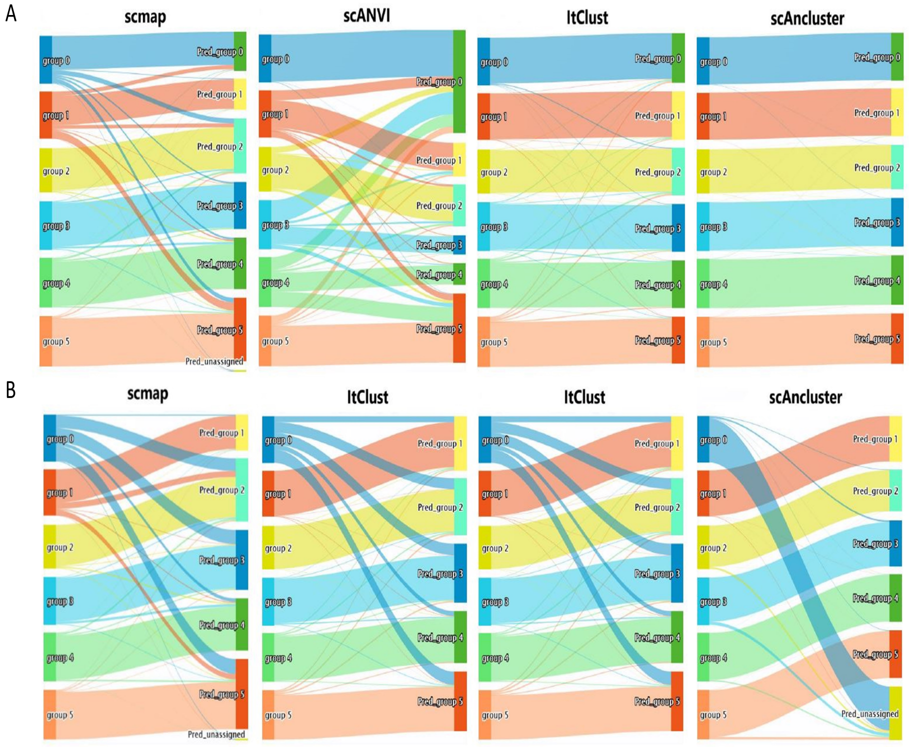

Fig. 6: Simulation dataset analysis. (A) Sankey plots for four methods on one mixed complete dataset. (B) Sankey plots for four methods on one mixed dataset that removes group 0 in the source dataset.

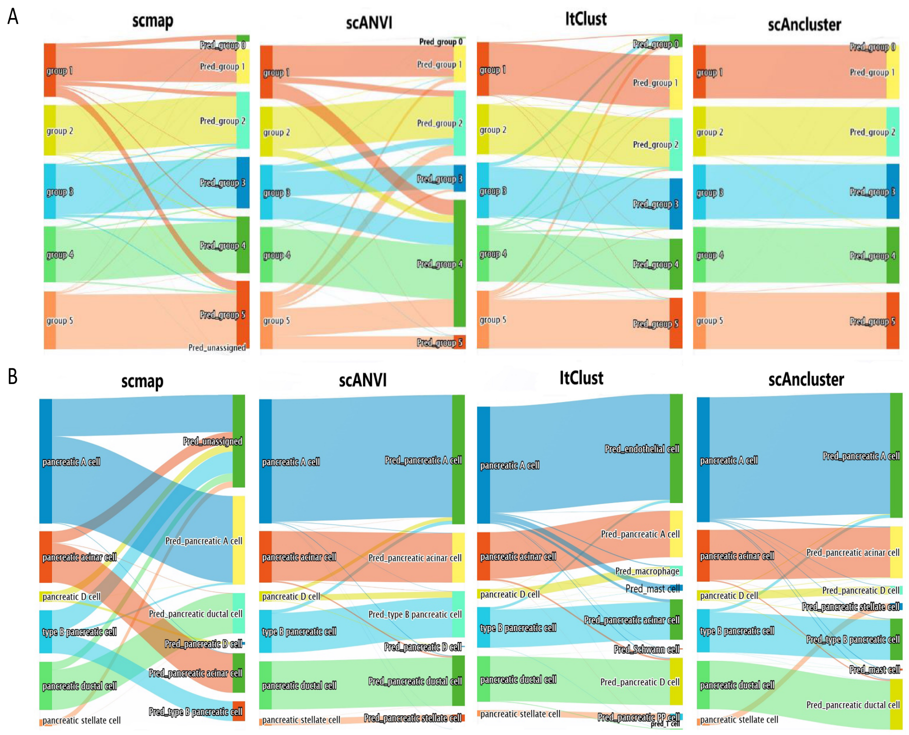

Fig. 7: Simulation and real dataset analysis. (A) Sankey plots for four methods on one mixed dataset that removes group 0 in the target dataset. (B) Sankey plots for four methods on the whole "Baron_human+Enge" dataset.

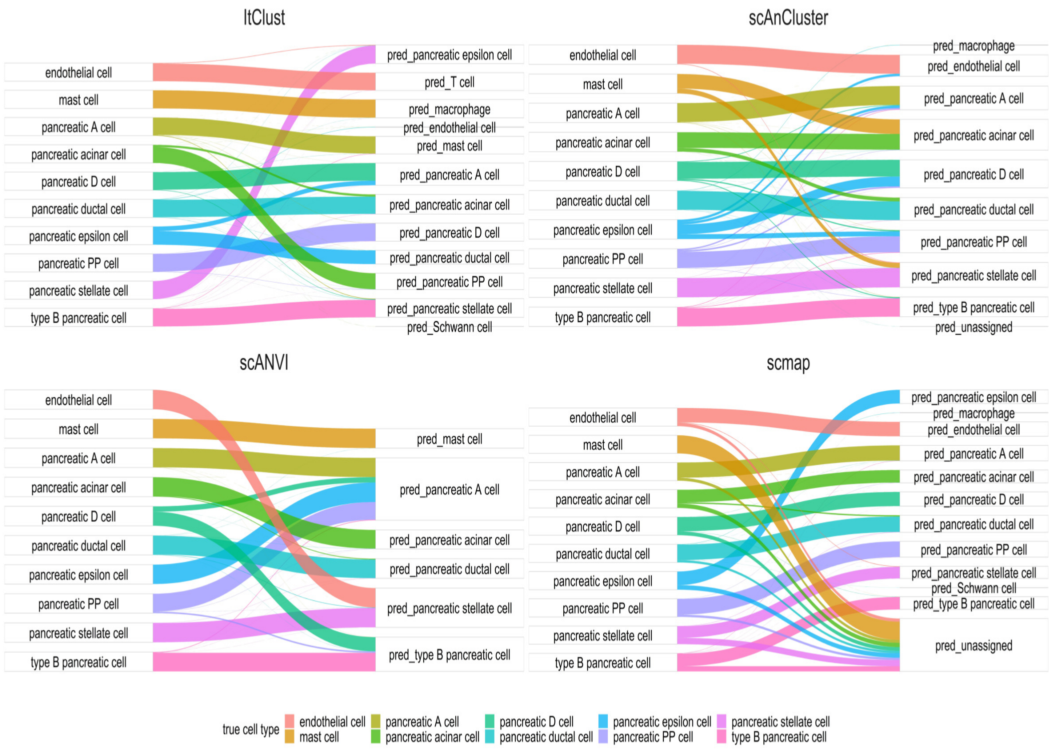

Fig. 8: Real dataset analysis. Sankey plots for four methods on the whole "Baron_human+Enge+Muraro+Segerstolpe+Xin" dataset. The upper row represents ItClust and scAnCluster, and the lower row represents scANVI and scmap.

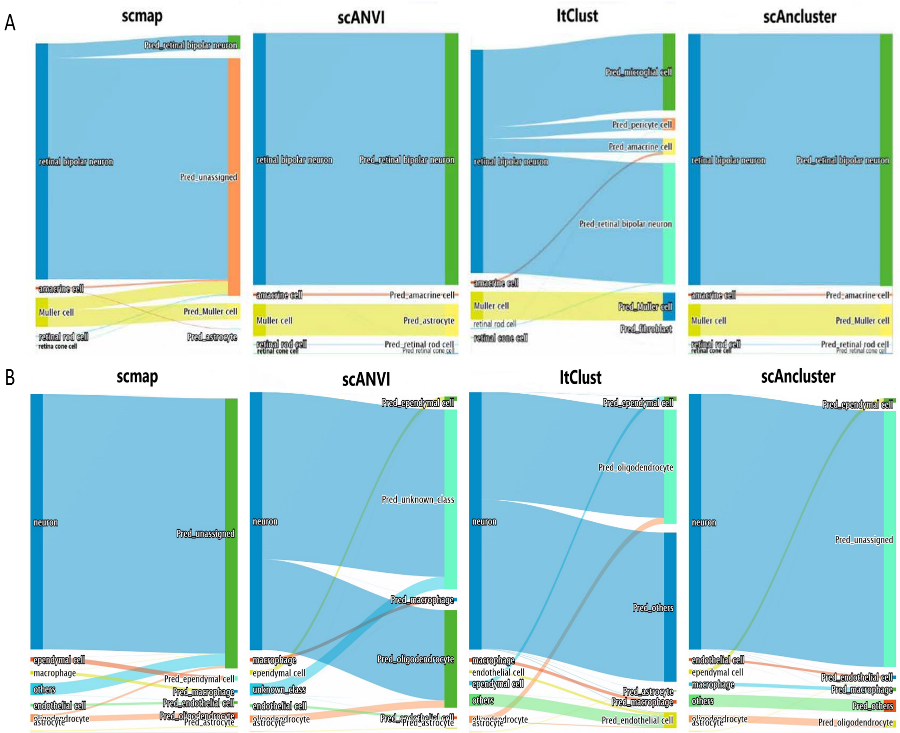

Fig. 9: Real dataset analysis. (A) Sankey plots for four methods on the whole "Macosko+Shekhar" dataset. (B) Sankey plots for four methods in the "large(c)" experiment on the "Campbell" dataset that automatically divides all neurons into the target dataset.
